# Evaluation of the Therapeutic Outcomes of Antibiotic Regimen Against Carbapenemase-Producing *Klebsiella pneumoniae*: A Systematic Review and Meta-Analysis

**DOI:** 10.3389/fphar.2021.597907

**Published:** 2021-11-04

**Authors:** Clement Yaw Effah, Emmanuel Kwateng Drokow, Clement Agboyibor, Shaohua Liu, Emmanuel Nuamah, Tongwen Sun, Lijun Miao, Jing Wang, Zhiwei Xu, Yongjun Wu, Xiaoju Zhang

**Affiliations:** ^1^ College of Public Health, Zhengzhou University, Zhengzhou, China; ^2^ Department of Radiation Oncology, Henan Provincial People’s Hospital, Zhengzhou University People’s Hospital, Zhengzhou, China; ^3^ School of Pharmaceutical Sciences, Zhengzhou University, Zhengzhou, China; ^4^ General ICU, Henan Key Laboratory of Critical Care Medicine, The First Affiliated Hospital of Zhengzhou University, Zhengzhou, China; ^5^ College of Agriculture and Natural Sciences, University of Cape Coast, Cape Coast, Ghana; ^6^ Department of Respiratory and Critical Care Medicine, Henan Provincial People’s Hospital & People’s Hospital of Zhengzhou University, Zhengzhou, China

**Keywords:** *Klebsiella pneumoniae*, carbapenemase-producing, clinical outcomes, meta-analysis, antibiotic regimen

## Abstract

**Background:** Carbapenemase-producing *Klebsiella pneumoniae* (CpKP) has been implicated as an increasing threat to public health. CpKP is a ubiquitous, opportunistic pathogen that causes both hospital and community acquired infections. This organism hydrolyzes carbapenems and other β-lactams and thus, leading to multiple resistance to these antibiotics. Despite the difficult to treat nature of infections caused by CpKP, little has been discussed on the mortality, clinical response and microbiological success rates associated with various antibiotic regimen against CpKP. This meta-analysis was designed to fill the paucity of information on the clinical impact of various antibiotic therapeutic regimens among patients infected with CpKP.

**Materials and Methods:** Literature in most English databases such as Medline through PubMed, Google Scholar, Web of Science, Cochrane Library and EMBASE, were searched for most studies published between the years 2015–2020. Data were analyzed using the R studio 2.15.2 statistical software program (metaphor and meta Package, Version 2) by random-effects (DerSimonian and Laird) model.

**Results:** Twenty-one (21) studies including 2841 patients who had been infected with CpKP were analysed. The overall mortality rate was 32.2% (95%*CI* = 26.23–38.87; *I* ^*2*^ = 89%; *p*-value ≤ 0.01, Number of patients = 2716). Pooled clinical and microbiological success rates were 67.6% (95%*CI* = 58.35–75.64, *I* ^*2*^ = 22%, *p*-value = 0.25, Number of patients = 171) and 74.9% (95%*CI* = 59.02–86.09, *I* ^*2*^ = 53%, *p*-value = 0.05, Number of patients = 121), respectively. CpKP infected patients treated with combination therapy are less likely to die as compared to those treated with monotherapy (OR = 0.55, 95%*CI* = 0.35–0.87, *p*-value = 0.01, Number of patients = 1,475). No significant difference existed between the mortality rate among 60years and above patients vs below 60years (OR = 0.84, 95%*CI* = 0.28–2.57, *p*-value = 0.76, 6 studies, Number of patients = 1,688), and among patients treated with triple therapy vs. double therapy (OR = 0.50, 95%*CI* = 0.21–1.22, *p*-value = 0.13, 2 studies, Number of patients = 102). When compared with aminoglycoside-sparing therapies, aminoglycoside-containing therapies had positive significant outcomes on both mortality and microbiological success rates.

**Conclusion:** New effective therapies are urgently needed to help fight infections caused by this organism. The effective use of various therapeutic options and the strict implementation of infection control measures are of utmost importance in order to prevent infections caused by CpKP. Strict national or international implementation of infection control measures and treatment guidelines will help improve healthcare, and equip governments and communities to respond to and prevent the spread of infectious diseases caused by CpKP.

## Introduction

*Klebsiella pneumoniae* is one of the *Enterobacteriaceae* that has increasingly become a threat to public health (Effah et al., 2020), and has been implicated in severe mortality and morbidity (Munoz-Price et al., 2013; World Health Organization, 2017). *K. pneumoniae* is a ubiquitous, opportunistic pathogen that causes both hospital and community acquired infections including pneumonia, urinary tract infection, bloodstream infections, cystitis, surgical wound infections, endocarditis, pyogenic liver abscesses and endogenous endophthalmitis (Podschun and Ullmann, 1998; Navon-Venezia et al., 2017). Based on several resistant mechanisms employed by this organism, there have been several strains that have been reported circulating worldwide. These mechanisms which include but not limited to vertical and horizontal transfer of resistant genes, efflux pump mechanisms and the dissemination and acquisition of resistant genes that are carried on plasmids and transposons have resulted in these hard to treat strains. The most notable strain of *K. pneumoniae* is the type that produces extended-spectrum β-lactamases (ESBLs). ESBL-*K. pneumoniae* (ESBL-KP) are now distributed worldwide and has been implicated in numerous outbreaks (Paterson and Yu, 1999; Tumbarello et al., 2006). According to Beigverdi et al. (2019), the pooled prevalence of ESBL-KP was 43.5% (95% CI 39.3–47.9%) among clinical *K. pneumoniae* isolates in Iran and the prevalence of each resistant mediated gene varies. Carbapenems are usually the antibiotics that are the first call or the first-line therapy that are employed during the treatment of severe infections caused by ESBL-producing *K. pneumoniae* (Pitout and Laupland, 2008). However, Yigit et al. (2001), Jacob et al. (2013) and other authors have reported on *K. pneumoniae* carbapenemase (KPC)-producing *K. pneumoniae* isolate. Carbapenemases are lactamases that hydrolyze carbapenems and other β-lactams and thus, provides resistance to these antibiotics. The Carbapenemase genes have since 2001 being spreading to all corners of the world. The KPC gene is the most common and the most prevalent of all the carbapenemases followed by the OXA genes (mainly bla_OXA-48_, bla_OXA-162_, and bla_OXA-181_), NDM genes, VIM genes, IMP genes, SME genes and others. The spread of these encoding plasmids poses a significant threat to public health as their acquisition leads to a multi-drug resistant (MDR) and extremely drug resistant (XDR) pathogen. In terms of epidemiological data, the prevalence of carbapenemase-producing *Enterobacteriaceae* (CPE) vary considerably from country to country. KPC and other carbapenemases including IPM, VIM, OXA-48 and NDM are known to be endemic in Israel, Japan, Greece, Turkey and India, respectively, and have been distributed across the globe (Cantón et al., 2012). KPC producers are also known to be endemic in some areas of Latin America, such as Colombia and Argentina (Levy Hara et al., 2013). There have been reported imports of KPC producers from endemic areas into Europe and this has led to a widespread of KPC producers to almost every part of Europe (Munoz-Price et al., 2013). European countries with the highest KPC-producing isolates are Italy and Greece, with rates ranging between 25 and 50% and >50% for invasive infections caused by CPE (European Antimicrobial Resistance Surveillance Network (EARS-Net), 2017). In Israel, KPC producers have been linked to numerous healthcare-associated reports and some community-acquired cases. The extent to which KPC producers are distributed in South East Asia is unknown, even though China may face some endemic situations. Reports on KPC-producing isolates in India are scanty, however, NDM and OXA-48-like carbapenemases are the most commonly reported. ST 258, a *K. pneumoniae* clone that produces KPC-2 or KPC-3, has been widely identified worldwide (Cuzon et al., 2010). Several outbreaks of different NDM producers have been documented in Pakistan, India and Sri Lanka (Poirel et al., 2011). In India, the prevalence of NDM is estimated at 5–15% (Day et al., 2013). Because of their close ties with India and Pakistan, the United Kingdom (UK), has recorded considerable spread of NDM producers (Johnson and Woodford, 2013). Since then, there have been a number of reports on NDM producers in *Enterobacteriaceae* from countries all over the globe, including countries in Africa, Asia, Europe, Australia, and the Americas (Berrazeg et al., 2014). OXA-48 producers have been extensively reported in Turkey, North Africa, the Middle East and India as the cause of healthcare-associated outbreaks (European Antimicrobial Resistance Surveillance Network (EARS-Net), 2017; Girlich et al., 2014). There is a growing prevalence of this carbapenemase in many countries of Europe, including France. As reported by the European Antimicrobial Resistance Surveillance Network (EARS-Net), 2017, carbapenem resistance was rampantly recorded in *K. pneumoniae* between the years 2014 and 2018, with some countries reporting resistance rates exceeding 10%.

Due to the difficult to treat nature of infections caused by carbapenem-producing *K. pneumoniae* (CpKP), there are limited treatment options that are currently available. Studies have reported that antibiotics such as colistin, gentamicin, tigecycline, and fosfomycin that are used for the treatment of infections caused by CpKP can lead to severe adverse effects such as nephrotoxicity (Akajagbor et al., 2013; Osorio et al., 2017) and hepatotoxicity (Lim et al., 2010; Florescu et al., 2012). According to (Tumbarello et al., 2012a; Daikos et al., 2014), it has been reported that during bacteremic infections, combination treatment regimens are recommended; however, it has been reported by Gutiérrez-Gutiérrez et al. (2017) that, combination therapies should be used when patients are in critical conditions. Also, in some retrospective studies on bloodstream infections caused by CpKP, it was realized that patients that were placed on antibiotic combination regimen were significantly more likely to survive than patients who were on monotherapeutic regimen (Zarkotou et al., 2011; Qureshi et al., 2012). Although a lot of studies have asserted that the use of more than one *in vitro* active drug may be superior to monotherapy, the rate of clinical failure still remains high (Tzouvelekis et al., 2014; Tumbarello et al., 2015a; Gutiérrez-Gutiérrez et al., 2017).

Despite the milestones achieved by various researchers in their quest to add to knowledge on the mortality, clinical response and microbiological response rates associated with various antibiotic regimen against carbapenemase-producing *K. pneumoniae*, there is still a paucity of information that needs to be filled. Most of the available review studies have focused on the mortality outcomes associated with these regimens (Tzouvelekis et al., 2014; Xu et al., 2017; Rodríguez-Baño et al., 2018) and these studies have not focused on CpKP but rather the broader carbapenem-resistant *Enterobacteriaceae* (CRE). To know the true effect of these therapeutic regimens, there is a need to evaluate the total clinical outcomes (mortality rates, clinical success rate and the microbiological success rates) associated with these regimens.

In this current study, we reviewed the clinical impact of various antibiotic therapeutic regimen against CpKP infected patients. First, we reviewed on various mono and combination therapies utilized in the treatment of infections caused by CpKP. Then, we finally performed a meta-analysis to evaluate the therapeutic outcomes of various antibiotic regimen on various clinical outcomes.

### Current and Emerging Treatment Strategies Against CpKP

#### Monotherapy

The most active aminoglycoside against CpKP is gentamicin (Castanheira et al., 2010; Souli et al., 2010; Livermore et al., 2011). There are limited data on the use of aminoglycosides as monotherapy, however, aminoglycoside monotherapy appears to be the most effective in the treatment of urinary tract infections (UTIs) caused by CpKP (Hirsch and Tam, 2010; Satlin et al., 2011; Tzouvelekis et al., 2014). When compared to the use of polymyxin B or tigecycline in the treatment of CpKP bacteriuria, treatment with an *in vitro* active aminoglycoside resulted in a much greater rate of microbiological clearance (Satlin et al., 2011). The use of targeted gentamicin among CpKP-induced sepsis patients was associated with reduced mortality (20.7 versus 61.9%, *p* = 0.02) until Day 30 (Gonzalez-Padilla et al., 2015). Also, oral gentamicin treatment was effective in the eradication of CpKP from the gastrointestinal reservoir which means that gentamycin could serve as additional tool in the combat against the nosocomial spread and severe infections caused by this difficult-to-treat organism (Zuckerman et al., 2011).

In a study by Lee et al. (2009), blood isolates from one patient infected with CpKP and treated with polymyxin B monotherapy showed a significantly increased polymyxin B MIC in just 5 days (0.75 g/ml to 1,024 g/ml). A meta-analysis of 15 studies involving 55 different patients concluded that colistin monotherapy had lower clinical success than colistin combination therapy (with tigecycline or gentamicin) for the treatment of infections caused by CpKP (14.3% [1 of 7] vs. 72.7% [8 of 11]) (Hirsch and Tam, 2010).

In addition, a study involving a small number of patients infected with CpKP concluded that 71.4 percent (5 of 7) had a favourable clinical outcome with tigecycline treatment (Hirsch and Tam, 2010). In two separate cohort studies, high mortality rates were reported to be associated with the use of tigecycline monotherapy in the treatment of bloodstream infections caused by CpKP (Tumbarello et al., 2012b; Daikos et al., 2014). One study assessed the therapeutic outcomes of tigecycline on 164 non-duplicate clinical strains of CpKP isolated from hospital-acquired pneumonia (HAP). The study found that doubling the tigecycline dose resulted in a higher cumulative fraction of response, which is an indication of better clinical efficacy (90.2 percent vs. 71.2 percent) (Trecarichi et al., 2016). Balandin Moreno et al. (2014) discovered no statistical significant differences in mortality rates between high-dose tigecycline and standard-dose tigecycline.

Other potential monotherapies with promising outcomes include: cefiderocol, a new siderophore cephalosporin, which has shown significant *in vitro* and *in vivo* activity against CpKP (Saisho et al., 2018). Cefiderocol has a unique antibacterial mechanism in which its catechol side chain binds to ferric acid, and the complex is actively carried into bacteria via bacterial iron transporters (Ito et al., 2016). Cefiderocol is also very effective at inhibiting carbapenemase hydrolysis (Wright et al., 2017). Cefiderocol has shown considerable *in vitro* activity against carbapenem-resistant Enterobacteriaceae (CRE) isolates, recording a cefiderocol MICs of less than 4 mg/L in 97.0 percent (991/1,022) of isolates (Hackel et al., 2018). Eravacycline is another unique synthetic tetracycline analogue which overcomes the basic mechanism of tetracycline resistance and possesses *in vitro* action against CpKP. However, its clinical evidence on the treatment of CpKP is sparse. Apramycin is also a monotherapy with promising action against CpKP. Apramycin is an aminoglycoside that has been traditionally used in veterinary medicine.

#### Combination Regimens

Treatment with a dual-carbapenem may be an effective option for CpKP infections (Ceccarelli et al., 2013; Giamarellou et al., 2013). Based on experimental data, it has been known that the affinity of KPC enzyme for ertapenem is higher than other carbapenems; therefore, when administered together, KPC preferentially inactivates ertapenem, thereby preventing degradation and subsequently enhancing the potency of the other carbapenems (Bulik and Nicolau, 2011; Wiskirchen et al., 2013). According to case reports, ertapenem in combination with doripenem or meropenem has been used successfully to treat CpKP infections (bacteremia, ventilator-associated pneumonia, and UTI). Dual-carbapenem therapy is a promising option that may be most effective when used concomitantly with a third drug (Zavascki et al., 2013). To assess the mortality rate associated with carbapenem-containing-combination therapy for CpKp bacteremia, Daikos et al. (2014) performed a large cohort study and reported an increased mortality rate from 19.4% (6 of 31, MIC ≤8 μg/ml) to 35.5% (11 of 31, MIC >8 μg/ml). In a meta-analysis of 20 clinical studies, it was realized that therapies that contain carbapenem recorded lower mortality rates compared to the non-carbapenem-containing regimens (18.8 vs. 30.7%) (Tzouvelekis et al., 2014). In addition, a retrospective study from a 10-bed intensive care unit found that carbapenem-sparing-combination therapy was effective in 24 of 26 (92 percent) patients with CpKP infections (Sbrana et al., 2013). A non-controlled case series study found that combining two carbapenems (ertapenem plus prolonged infusion of meropenem or doripenem) can be effective in treating CpKP infections, with clinical success rates ranging from 39 to 77.8 percent (Cprek and Gallagher, 2016; Souli et al., 2017).

A new β-lactam/β-lactamase inhibitor (BL/BLI), ceftazidime/avibactam, has been approved for the treatment of complicated intra-abdominal infection, complicated urinary tract infection, hospital-acquired and ventilator-associated pneumonia caused by CpKP (Kaye and Pogue, 2015). Avibactam, unlike most β-lactamase inhibitors, is not a β-lactam. However, this novel drug is a unique synthetic non-β-lactam (diazabicyclooctane)/β-lactamase inhibitor that inhibits a wide range of β-lactamases, including Ambler Class A (GEM, SHV, CTX-M, and KPC), Class C (AmpC), and some Class D (OXA-48) β-lactamases (de Jonge et al., 2016). However, Class B metallo- β-lactamases (MBLs) (IMP, VIM, VEB, and NDM) are not inhibited by avibactam (Syue et al., 2016; Wong and van Duin, 2017). Because of this great inhibitory potential, avibactam, when added to ceftazidime, restores the potent activity of ceftazidime against CpKP and other CRE.

Furthermore, to achieve a more efficient synergistic effect in the treatment of infections caused by CpKP, meropenem/vaborbactam (MER-VAB) can be used alone or in combination with non-active *in vitro* drugs (Qureshi et al., 2012; Guimarães et al., 2019; Tumbarello et al., 2019). Because Ambler class A and C carbapenemases are responsible for conferring resistance to meropenem by most bacteria, vaborbactam, which is a novel boron-containing serine lactamase inhibitor, has been proposed as a suitable option for restoring the efficacy of meropenem against organisms possessing these enzymes (Castanheira et al., 2017). However, it has no *in vitro* activity against Class B MBLs producers (NDM or VIM) or Class D OXA-48 β-lactamases (Castanheira et al., 2016; Nelson et al., 2017). The development of resistance to MER-VAB by CpKp is rarely documented. However, when it happens, it may be as a result of mutation in ompK35 and ompK36 or, mutation of kvrA which leads to downregulation of the ompK35/36 porin. Furthermore, when compared to CAZ/AVI, MER-VAB is less affected by KPC-2 mutations which confers resistance to CAZ/AVI. Base on this, it can be argued that MER-VAB offers the best therapeutic option available for CpKP.

Imipenem/cilastatin and relebactam therapy is one of the potent therapies against CpKP, and is made up of a combination of an approved carbapenem and a novel BLI. The chemical structure of relebactam is similar to that of avibactam (Watkins et al., 2013). The structure is made up of a diazabicyclooctane core, which under *in vitro* conditions, covalently and reversibly binds to Class A and C β-lactamases. Relebactam also possesses similar inhibitory mechanism as avibactam (Blizzard et al., 2014). Just like avibactam, relebactam cannot inhibit Class D OXA-48 (Petty et al., 2018; Zhanel et al., 2018).

Cefepime/zidebactam is one promising therapy which is made up of a combination of cefepime and the novel BLI, zidebactam. Unlike the other BLI, Zidebactam possess dual inhibitory mechanisms; it has high-affinity to penicillin binding protein 2 (PBP2) and also inhibit Ambler class A and C carbapenemase enzymes. When compared to the use of cefepime alone, the combination of zidebactam with cefepime has been shown to be a more effective therapy against CpKp (Livermore et al., 2017; Thomson et al., 2019). Other promising and emerging BL/BLI combination therapy against CpKP are Cefepime/taniborbactam, Imipenem/relebactam, Meropenem/nacubactam and Meropenem/QPX7728 combination.

## Materials and Methods

This study followed the required guidelines and reporting procedures for Systematic Reviews and Meta-analysis (PRISMA) (Moher et al., 2009).

### Search Strategy

The literature search was conducted in most English databases such as Medline through PubMed, Web of Science, Cochrane Library, EMBASE, and Google scholar. The search was carried out by three independent individuals. The keywords employed in the search were Klebsiella pneumoniae*, carbapenem-producing*, antibiotic regimens*, clinical outcomes*, mortality rate*, clinical success*, microbiological success*, carbapenem resistance*, VIM-producing*, IMP-producing*, NDM-producing*, Metallo beta lactamase*, OXA*, KPC*, CRE* and the search was restricted to studies published between the years 2015–2020.

### Inclusion and Exclusion Strategy

For a study to meet the inclusion criteria of this study, it should have reported the outcomes among CpKP infected patients following antibiotic regimes. Prospective and retrospective randomized-controlled trials (RCTs), cohort and case control studies as well as case series and case reports were included. Studies were originally screened for eligibility in this review based on their titles and abstracts. Eligible studies were chosen in three steps: first, on the basis of the title, then on the basis of the abstract, and finally, on the basis of the full-text publication. Studies were deemed suitable for this work if clinical outcomes such as clinical response, microbiological response and mortality had been reported; if the phenotypic detection of the carbapenemase genes was done by methods such as Etest, or doubledisk synergy test based on the guidelines provided by the Clinical and Laboratory Standards Institute, 2015; and if molecular methods such as PCR were used in the confirmation and detection of the carbapenemase gene. Studies that were excluded from this study were, conference proceedings, editorials, duplicate publications, animal and *in vitro* studies.

### Data Extraction Methods

For this meta-analysis, the following data were extracted from the various studies; the name of the first author, the type of Study, the year of publication, the country where the study was conducted, the study period, sample size, study characteristics (such as the site of infection, age of patients, comorbidities, etc), the susceptibility breakpoints standards used and clinical outcomes (such as mortality, clinical success, microbiological success).

### Study Quality Assessment

Three reviewers assessed the quality of the studies base on the quality assessment scale. Any disagreements among the reviewers were resolved through dialogue. For this meta-analysis, the validity of the non-randomized studies was assessed with the Newcastle-Ottawa Scale (NOS) for non-randomized studies (Wells, 2004); for a study to be included, a score of 5 or more was needed.

### Definitions and Outcomes

The definitions of outcomes that were used in this meta-analysis were those retrieved from individual studies. Crude mortality was defined as death by any cause evaluated at end of patient follow-up. Clinical success was defined as resolution of clinical signs and symptoms of the infections without relapse at the end of the antibiotic treatment. The microbiological success was defined as a culture negative for CpKP from subsequent specimen cultures. Different studies had different experimental durations. Generally, Clinical and microbiological outcome among studies was evaluated on D14 and D28 (some studies at the end of D30 or D90). Microbiological success was defined as a culture negative at D14 and/or D28. Follow-up also vary among studies and in some studies this was possible until discharge from the hospital or death. Mortality was evaluated at the end of the follow-up period (i.e. either D14, D28, D30 or D90).

### Statistical Analysis

For the overall microbiological response, clinical success and mortality estimation, the Freeman-Tukey double arcsine transformed proportions and the random-effects (DerSimonian-Laird) model (Borenstein et al., 2009) were used. Following antibiotic therapies, the treatment outcomes were compared and the results were presented as odds ratios [OR]. Subgroup and sensitivity analyses were performed according to the various treatment regimens. Q test was used to evaluate between-study heterogeneity. The reliability of pooled assessments was evaluated via the leave-1-out sensitivity analyzes, and a study was considered relevant when the pooled result without that study, was beyond the 95% confidence interval [*CI*] of the overall pooled estimate. The degree of heterogeneity was quantified by *I* ^*2*^ with the significance level set at 0.05. A high *I* ^*2*^ value (>50%) indicated heterogeneity. If heterogeneity was not present (*I* ^*2*^ < 50%), a fixed-effect model was applied for analysis; otherwise, a random effect model was adopted. The visualization approach of the funnel plots was utilized in the assessment of publication bias whilst the Egger’s test was used to quantify this bias. Statistical analyses were performed using the R studio 2.15.2 statistical software program (metaphor and meta Package, Version 2) by random-effects (DerSimonian and Laird) model.

## Results

### Systematic Literature Review and Study Characteristics

**FIGURE 1:**
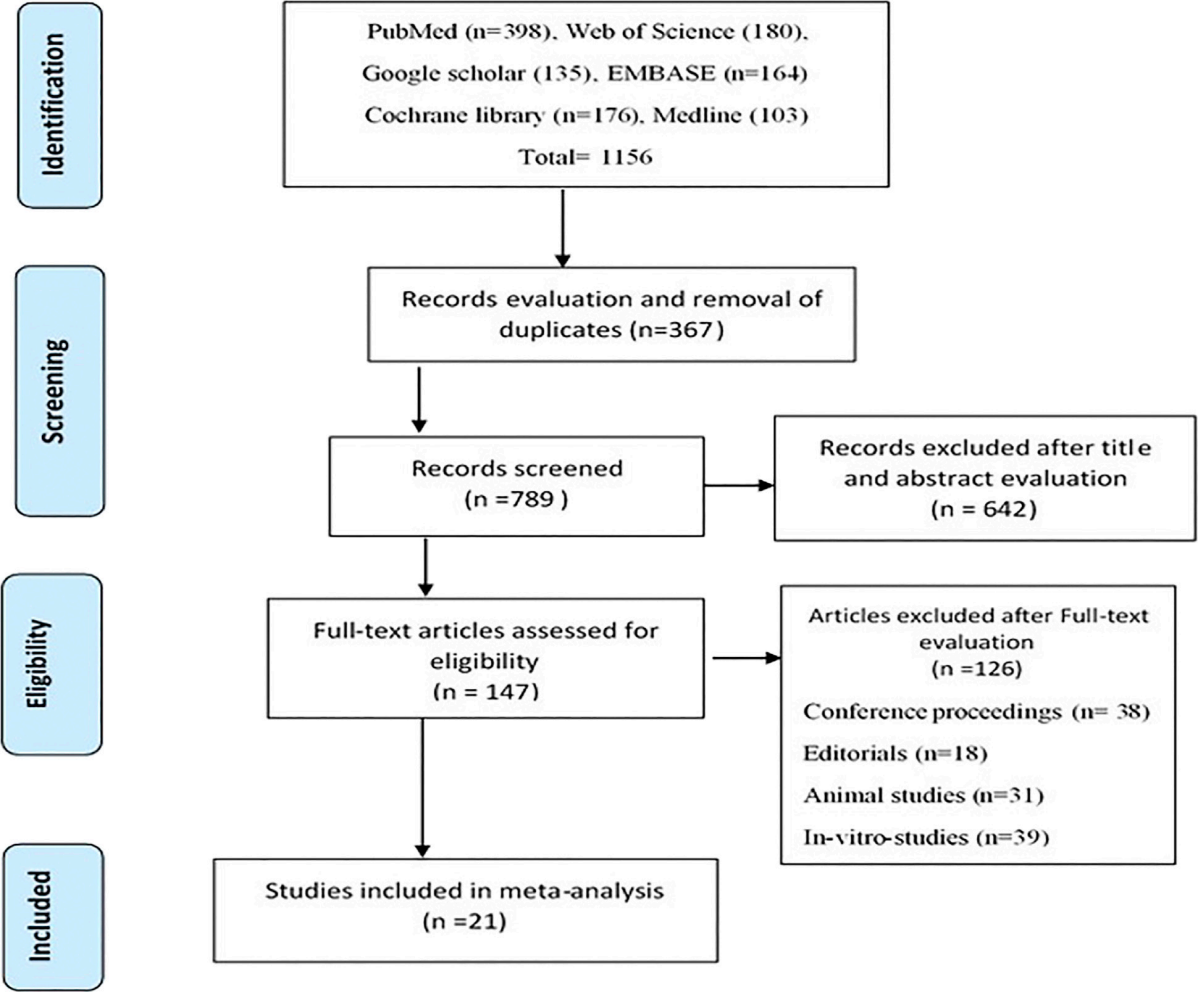
Prisma flow chart of study selection.

After an extensive literature search, 1,156 studies were initially retrieved. After record evaluation and assessment for duplicates, 367 references were excluded. Six hundred and forty-two (642) studies were further eliminated after the study titles and abstracts were evaluated. The Full-text assessment was carried out on 147 studies, of which 126 studies were further eliminated. The primary reasons for rejection were that the articles were conference proceedings (38), editorials (18), animal studies (31) and *in vitro* studies (39). Twenty-one (21) studies met the inclusion criteria set for this meta-analysis. The Prisma flow chat (Figure 1) gives a detailed screening and selection procedure of this study.

Table 1 describes and summarizes the basic characteristics of the studies included in this meta-analysis. These studies were made up of 2841 patients who had been infected by CpKP. The average age reported in these studies ranges from 46 to 69years. Comorbidities associated with these patients were urinary tract infections, bloodstream infections, pneumonia, diabetes, cancer, chronic renal failure and cardiovascular disease among others. Also, the bloodstream, lungs, urinary tract, skin and soft tissues were the common sites of infection. Based on the Newcastle-Ottawa scale for nonrandomized trials scores of the included studies, studies were rated as having good quality (Table 2).

**TABLE 1:** Charcteristics of included studies.

Author (Year)	Study type (country)	Study period	Sample size	Site of infection	Susceptibility breakpoints used	Study characteristics	Age	Monotherapy outcome/mortality	Combination therapy outcome/mortality			Clinical success	Microbiological success
									Overall	Double combination	Tripple combination		
Gregory (2019)	SC retrospective, Brazil	2015–2016	82	BSI (13%), pneumonia (30.5%), UTI (11%), SSI (11%)	CLSI (2017), EUCAST (2017) for colistin and tigecycline	Cardiovascular disease (47.6%), Cancer (40.2%), Chronic lung disease (15.9%), Chronic kidney disease (29.3), HIV (7.3)	57.6 ± 17 (mean ± SD)	26/36	17/45	NA	NA	NA	NA
Shaw et al. (2018)	SC retrospective, Spain	2016–2017	10	cUTI (20%), Pneumonia (20%), BSI (10%)	EUCAST (2017)	oesophageal cancer (10%), cirrhosis (10%), kidney stones (20%), prostate cancer (10%), Liver transplant (30%)	68.5 (mean)	NA	3/10	NA	NA	6/10	NA
Machuca et al. (2017)	SC prospective, (Spain)	2012–2016	104	BSI, UTI, pneumonia	EUCAST (2000), US FDA (for tigecycline)	All inpatients, chronic renal disease (26%), baseline renal failure (42.3%), diabetes (34.6%), COPD (14.4%), transplant (10.6%), active solid tumour (28.8%), septic shock (46.2%), critical care (53.8%)	NA	14/32	18/72	12/40	6/32	NA	NA
Gutiérrez-Gutiérrez et al. (2017)	MC retrospective, Multi-country	2004–2013	375	BSI (100%)	CLSI (2012)	NA	NA	85/208	47/135	NA	NA	NA	NA
Giannella et al. (2018)	MC prospective, Italy	2010–2015	595	BSI (100%)	EUCAST	NA	65.5 (median), 54–76 (IQR)	127/595	NA	NA	NA	NA	NA
Alexandra (2016)	NA	NA	15	UTI (8), SSTI (2), EPI (2), PNA (1), MSI (2)	NA	NA	60.9 ± 10.9 (mean ± SD)	NA	NA	NA	NA	12/15	12/15
Tanır Basaranoglu et al. (2017)	NA	NA		SBSI (1), CRBSI (2)	NA	NA	<1year	NA	NA	NA	NA	2/3	3/3
Rosa (2018)	NA	NA	2	UIT	NA	NA	46 (mean)	NA	NA	NA	NA	2/2	2/2
De Oliveira et al. (2015)	MC Retrospective	NA	118	NA	NA	NA	NA	21/57	32/61	NA	NA	NA	NA
Tumbarell et al. (2015a)	MC retrospective, Italy	2010–2013	661	BSI (67.6%), LRTI (12.9%), IAI (6.4%), UTI (12.4%), other (0.8%)	EUCAST (2015)	COPD (16%), CVD (41.6%), diabetes (25.4%), cerebrovascular disease or dementia (12.2%), haematological malignancy (13.5%), solid tumour (22.2%), liver disease (10.9%), chronic renal failure (18.4%), HIV (3%), neutropenia (10.6%), SOT (7.9%), shock (15.1%), ICU (34.8%)	68 (median) 55–76 (IQR)	80/156	93/291	NA	NA	NA	NA
Chang et al. (2015)	SC retrospective Autralia	2012	10	NA	NA	KPC-KP infected patients	NA	4/10	NA	NA	NA	NA	NA
Freire et al. (2015)	SC retrospective, Brazil	2009–2013	83	UTI (32.3%), BSI (38.9%), pneumonia (9.7%)	CLSI (2012), US FDA (for tigecycline), EUCAST (2014)	Oncology patients with culture positive for KPC-KP	54 (median) 21–72 (range)	49/83	NA	NA	NA	NA	NA
Venugopalan et al. (2017)	SC retrospective, USA	2010–2016	36	BSI (100%), UTI (41.7%), lung infection (22.2%), IAI (2.8%)	CLSI (2010)	CVD (86.1%), pulmonary disease (22.2%), diabetes (50%), malignancy (19.4%), CKD (27.8%), seizure disorder (8.3%)	NA	11/36	NA	NA	NA	13/18	15/16
Papadimitriou-Olivgeris et al. (2017)	SC retrospective, Greece	2012–2015	139	BSI (100%), pneumonia (0.7%), abdominal infection (2.9%)	EUCAST (2016)	ICU (100%), diabetes (18%), COPD (6.5%), chronic heart failure (12.2%), chronic renal failure (2.9%), malignancy (12.9%), septic shock (53.2%)	56.7 ± 18 (mean ± SD)	18/139	7/139	NA	NA	NA	NA
Liao et al. (2017)	SC retrospective, China	2012–2014	104	BSI (8.7%), pneumonia (81.7%), UTI (16.3%), IAI (3.8%)	NA	Diabetes (41.3%), COPD (27.9%), heart failure (23.1%), hepatic failure (1.9%), renal failure (23.1%), malignancy (13.5%), ICU (83.7%)	67.2 ± 15.7 (mean ± SD)	11/32	15/72	9/15	6/15	NA	NA
De Pascale et al. (2017)	MC retrospective, Italy	2012–2015	144	Pneumonia (51.4%), UTI (8.3%), BSI (57.6%), IAI (13.2%), SSTI (8.3%)	EUCAST	Chronic heart failure (31.3%), chronic renal failure (10.4%), COPD (16%), diabetes (33.3%), chronic liver disease (12.5%), septic shock (54.9%), polymicrobial (16%), ICU (100%)	59.4 (mean)	NA	42/144	NA	NA	30/48	22/44
Cprek and Gallagher, (2016)	SC retrospective, USA	2013–2014	18	BSI, pneumonia, UTI, SSSI, IAI	CLSI (2009)	CVD (56%), diabetes (44%), pulmonary disease (56%), immunocompromised state (39%), ICU (83%), polymicrobial (33.3%)	62.5 (median) 51–67 (IQR)	NA	5/18	NA	5/18	7/18	11/14
Oliva et al. (2016)	MC prospective, Italy	2012–2015	32	Pneumonia, EVD, Cuti, CRBI, BSI	NA	Septic shock (25%)	55.1 ± 15.2 (mean ± SD)	NA	6/32	6/32	NA	24/32	NA
Tumbarello et al. (2015b)	MC retrospective, Italy	2010–2013	8	BSI	EUCAST (2015)	NA	66 (median) 55–76 (IQR)	NA	3/8	3/8	NA	5/8	NA
Souli et al. (2017)	MC prospective, Greece	2012–2015	27	BSI, cUTI, VAP, EVD	CLSI (2012), EUCAST (2012) for fosfomycin, colistin, and tigecycline	Inpatients (100%) ICU (55.6%), cancer (7.4%), rheumatoid arthritis (7.4%), septic shock (22.2%)	59 (median) 15–83 (range)	NA	8/27	8/27	NA	21/27	20/27
Trecarichi et al. (2016)	MC prospective, Italy	2010–2014	278	BSI, UTI, respiratory tract infection	NA	Haematological malignancy (100%), diabetes (15.5%), chronic hepatic failure (1.8%), chronic renal failure (4.3%)	NA	NA	101/278	NA	NA	NA	NA

Abbreviations: MC, multicenter; SC, single center; BSI, bloodstream infection; UTI, urinary tract infection; ICU, intensive care unit; SSTI, skin and soft tissue infection; HIV, human immunodeficiency virus; CLSI, Clinical and Laboratory Standards Institute; EUCAST, European Committee on Antimicrobial Susceptibility Testing; IQR, interquartile range; SD, standard deviation; NA, not available; VAP, ventilator-associated pneumonia; COPD, chronic obstructive pulmonary disease; IAI, intraabdominal infection; LRTI, lower respiratory tract infection.

**TABLE 2:** Study quality assessment based on Newcastle-Ottawa scale.

Study	Quality assessment criteria								Total score
	Selection				Comparability	Outcome/Exposure			
	1	2	3	4		1	2	3	
Gregory (2019)	*	*	*	*	0	*	*		6
Shaw et al. (2018)	*	0	*	*	**	*	*	*	8
Machuca et al. (2017)	*	0	*	*	0	*	*	*	6
Gutiérrez-Gutiérrez et al. (2017)	*	*	0	*	0	*	*	0	5
Giannella et al. (2018)	*	0	*	*	0	*	*	0	5
Alexandra (2016)	*	*	0	*	0	0	*	*	5
Tanır Basaranoglu et al. (2017)	*	*	*	*	0	*	0	*	6
Rosa (2018)	*	*	*	*	0	*	*	0	6
De Oliveira et al. (2015)	*	0	*	*	0	*	*	*	6
Tumbarell et al. (2015a)	*	*	0	*	**	*	0	0	6
Chang et al. (2015)	*	*	*	*	0	*	0	0	5
Freire et al. (2015)	*	*	*	*	**	*	*	*	9
Venugopalan et al. (2017)	*	*	*	*	**	*	*	0	8
Papadimitriou-Olivgeris et al. (2017)	*	*	0	*	**	*	*	0	7
Liao et al. (2017)	*	0	*	*	0	*	0	*	5
De Pascale et al. (2017)	*	*	0	0	**	*	*	0	6
Cprek and Gallagher, (2016)	*	0	*	*	0	*	*	*	6
Oliva et al. (2016)	*	*	*	*	**	*	*	0	8
Tumbarello et al. (2015b)	*	0	*	*	0	*	*	*	6
Souli et al. (2017)	*	0	*	*	0	*	*	*	6
Trecarichi et al. (2016)	*	*	*	*	**	*	*	*	9

### Quantitative Synthesis

#### Overall Outcomes

**FIGURE 2:**
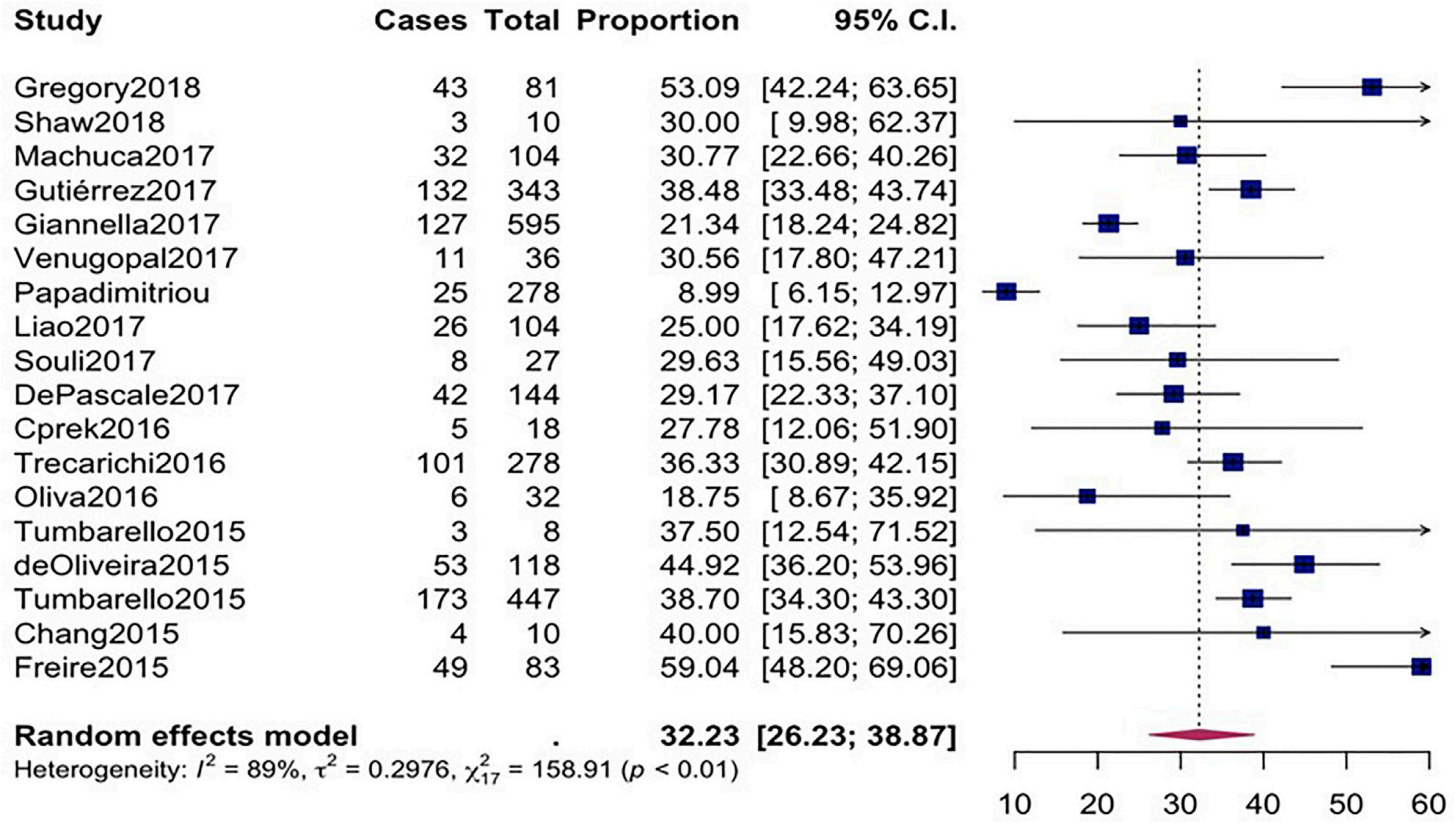
Forest plot for pooled mortality rate of CpKP infected patients.

**FIGURE 3:**
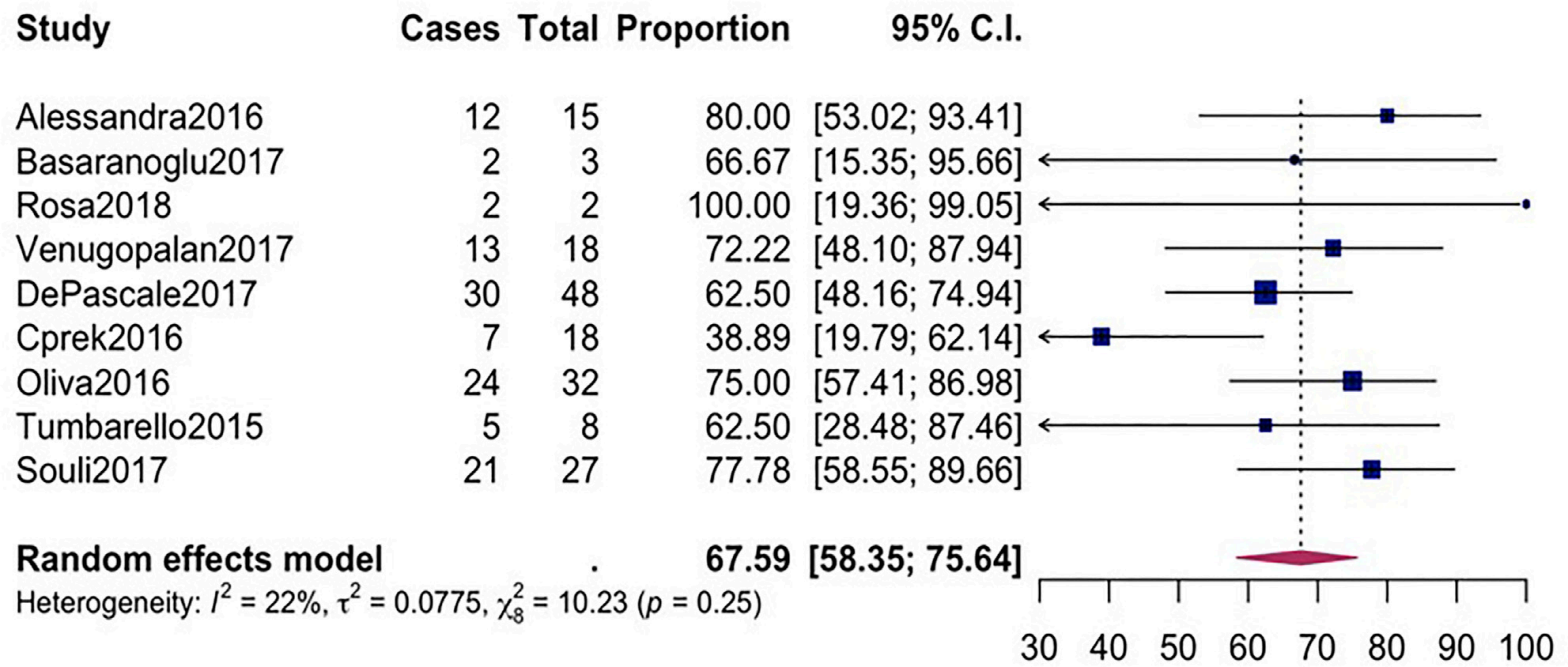
Forest plot for pooled clinical success among CpKP infected patients after antibiotic regimes.

**FIGURE 4:**
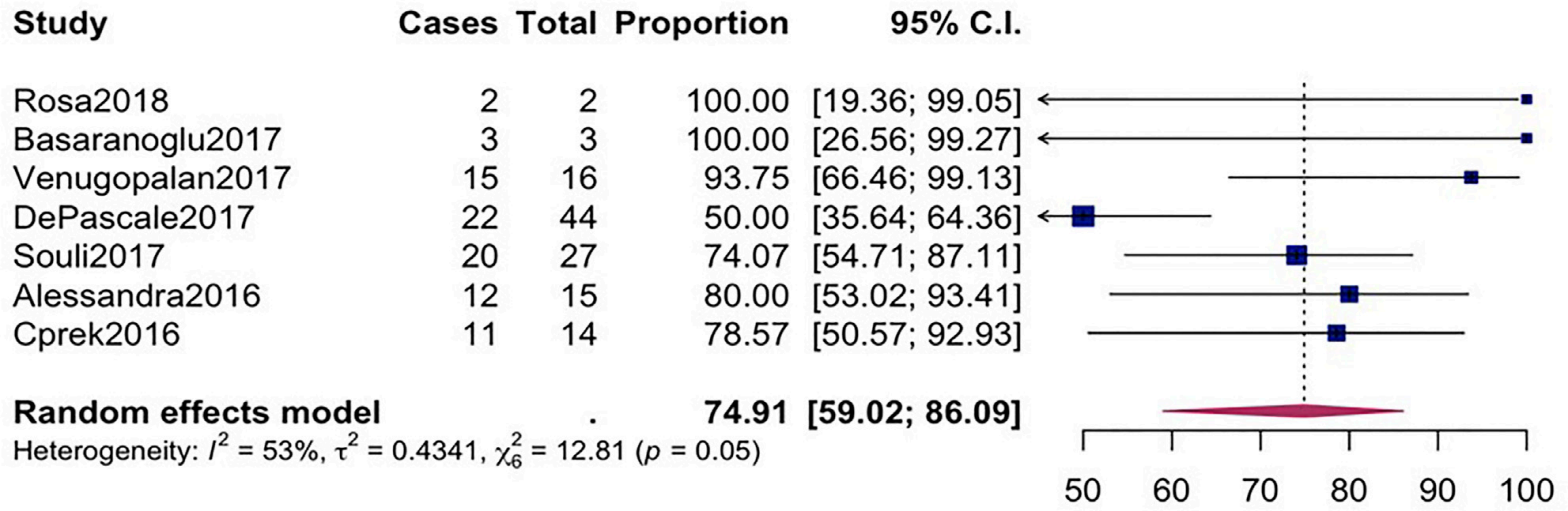
Forest plot for pooled microbiological success among CpKP infected patients after antibiotic.

**FIGURE 5:**
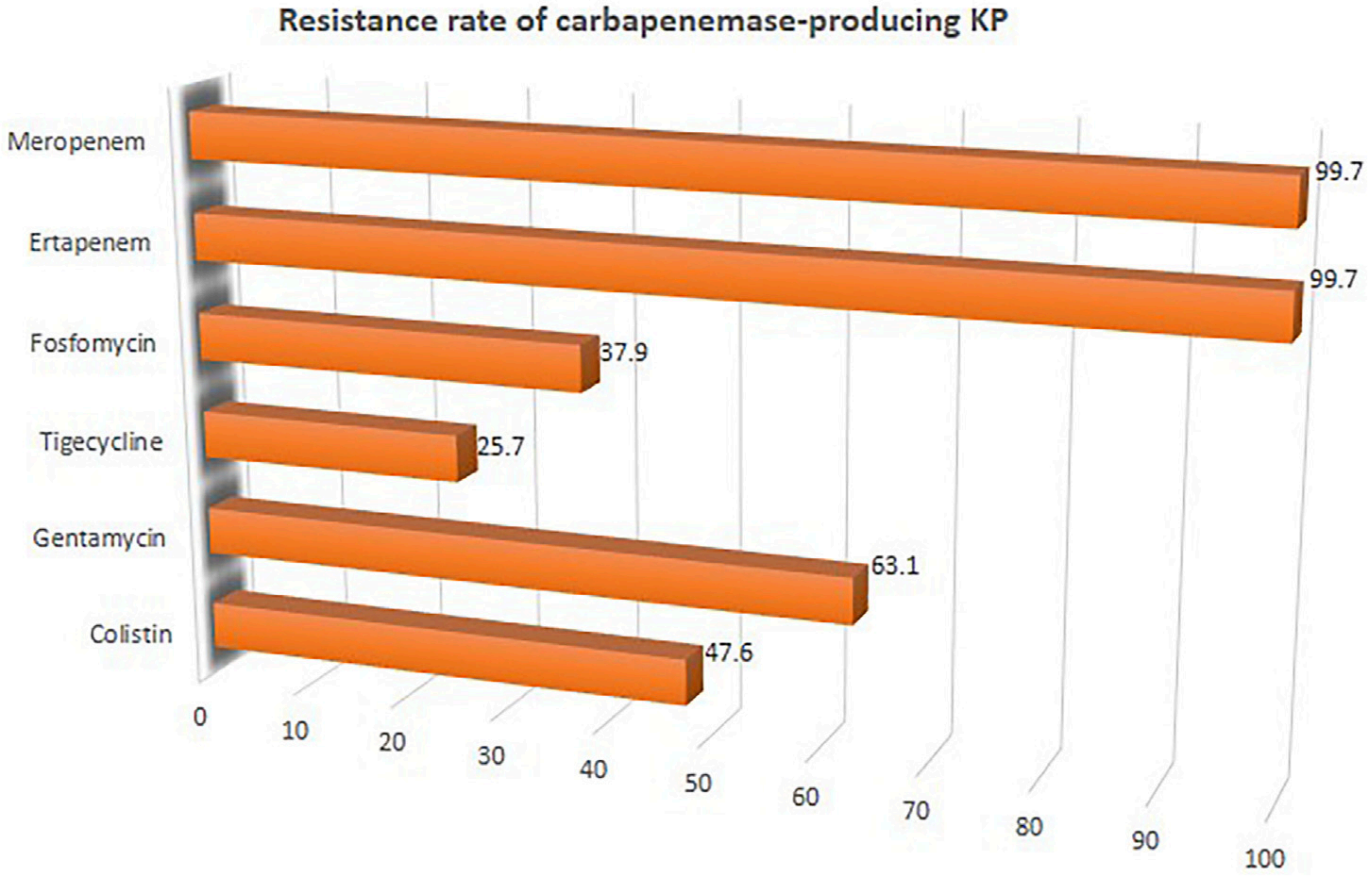
Resistant rate of CpKP to commonly used antibiotics in the treatment of CpKP infections. Colistin (*n* = 178), Gentamycin (*n* = 236), Tigecycline (*n* = 96), Fosfomycin (*n* = 89), Ertapenem (*n* = 373), Meropenem (*n* = 373).

The overall mortality rate from 15 studies involving 2716 patients that had received different antibiotic regimes was 32.2% (95%*CI* = 26.23–38.87; *I* ^*2*^ = 89%; *p*-value ≤ 0.01) (Figure 2). Pooled clinical success was estimated in 9 studies involving 171 CpKP infected patients treated with various antibiotic regimes (Figure 3). The overall clinical success rate was 67.6% (95%*CI* = 58.35–75.64, *I* ^*2*^ = 22%, *p*-value = 0.25) as compared to the microbiological success rate of 74.9% (95%*CI* = 59.02–86.09, *I* ^*2*^ = 53%, *p*-value = 0.05) which was realized from 7 studies involving 121 CpKP infected patients that have been treated with different antibiotic regimen (Figure 4). From Figure 5, the resistant rate of CpKP isolates varied across the different types of antibiotics. The resistant rates were Colistin (47.6%), Gentamycin (63.1%), Tigecycline (25.7%), Fosfomycin (37.9%), Ertapenem (99.7%) and Meropenem (99.7%).

#### Subgroup Analysis on Mortality Outcomes

Subgroup analysis on mortality was performed to ascertain the mortality rate of CpKP-infected patients. The following subgroup analyses were performed on patients: 1) Treated with combination therapy vs monotherapy 2) Among patients who are 60 years and beyond vs. less than 60 years 3) Who received triple combination therapy vs. double combination therapy 4) Among multicenter studies vs single center studies 5) Among studies published between 2015–2016 vs. studies published in 2017 and beyond 6) Among studies conducted in Europe vs others and 7) Among prospective studies vs retrospective studies (Table 3). Also various mortality outcomes associated with specific antibiotic class were analyzed. There is a significant difference between the mortality rate among CpKP patients treated with monotherapy and those treated with combination therapy. Patients treated with combination therapy had reduced death rate (OR = 0.55, 95%*CI* = 0.35–0.87, *p*-value = 0.01, 7 studies, Number of patients = 1,475). No significant difference existed between the mortality rate among the following subgroups: 60years and above patients vs below 60years (OR = 0.84, 95%*CI* = 0.28–2.57, *p*-value = 0.76, 6 studies, Number of patients = 1,688), triple therapy vs double therapy (OR = 0.50, 95%*CI* = 0.21–1.22, *p*-value = 0.13, 2 studies, Number of patients = 102), multicenter vs single center studies (OR = 1.01, 95%*CI* = 0.47–2.16, *p*-value = 0.98, 7 studies, Number of patients = 2404), studies published in 2017 and beyond vs studies published below 2017 (OR = 0.70, 95%*CI* = 0.33–1.47, *p*-value = 0.35, 8 studies, Number of patients = 2406) and prospective vs retrospective studies (OR = 0.71, 95%*CI* = 0.55–1.08, *p*-value = 0.13, 5 studies, Number of patients = 1706). Comparing studies conducted in Europe to those conducted elsewhere, it was realized that those in Europe had lower mortality as compared to those conducted elsewhere (OR = 0.50, 95%*CI* = 0.34–0.74, *p*-value ≤ 0.01, 7 studies, Number of patients = 1899). Comparing the mortality rates among the various antimicrobial class revealed that, apart from carbapenem vs aminoglycosides (OR = 1.37, 95%*CI* = 0.74–2.98, *p*-value = 0.02, 7 studies, Number of patients = 1,310) there were no significant difference between the mortality rates of CpKP patients treated with polymyxin vs tigecycline (OR = 0.89, 95%*CI* = 0.33–2.21, *p*-value = 0.67, 6 studies, Number of patients = 1,167), carbapenem vs. polymyxin (OR = 0.67, 95%*CI* = 0.46–1.66, *p*-value = 0.25, 4 studies, Number of patients = 860), and carbapenem vs tigecycline (OR = 0.98, 95%*CI* = 0.28–2.87, *p*-value = 0.78, 4 studies, Number of patients = 951) (Table 3). Comparing various combination therapies as seen in Table 3, it can be deduced that aminoglycosides-containing regimen significantly decreased the mortality rates among patients infected with CpKP as compared with patients treated with aminoglycosides-sparing regimen (OR = 0.86, 95%*CI* = 0.35–1.13, *p*-value = 0.04, 5 studies, Number of patients = 913).

**TABLE 3:** Subgroup analysis on mortality outcomes.

Mortality among	Studies	Number of patients	Test of association			Test of heterogeneity		
			*OR*	95%*CI*	*p-value*	*I* ^*2*^_*(%)*_	*p-value*	*X* ^*2*^
Combination therapy vs. Monotherapy	7	1,475	0.55	0.35–0.87	0.01	67	0.005	18.33
60years and above vs. Below 60years	6	1,688	0.84	0.28–2.57	0.76	92	<0.001	66.33
Triple therapy vs. Double therapy	2	102	0.50	0.21–1.22	0.13	<25	0.84	0.04
Multi-center vs. Single center	7	2404	1.01	0.47–2.16	0.98	86	<0.001	42.78
Data published 2017 and beyond vs. Data published below 2017	8	2406	0.70	0.33–1.47	0.35	88	<0.001	59.65
Studies conducted in Europe vs. Others	7	1899	0.50	0.34–0.74	<0.001	35	0.16	9.23
Prospective study vs. Retrospective study	5	1706	0.77	0.55–1.08	0.13	<25	0.86	1.31
Polymyxin vs Tigecycline	6	1,167	0.89	0.33–2.21	0.67	84	<0.001	47.23
Carbapenem vs. Aminoglycoside	7	1,310	1.37	0.74–2.98	0.02	72	0.78	28.6
Carbapenem vs. Polymyxin	4	860	0.67	0.46–1.66	0.25	<25	0.37	15.69
Carbapenem vs. Tigecycline	4	951	0.98	0.28–2.87	0.78	89	0.42	37.97
Aminoglycoside-containing combination therapy vs. Aminoglycoside-sparing combination therapy vs	5	913	0.86	0.35–1.13	0.04	<25	0.64	29.98
Polymyxin-containing combination therapy vs. Polymyxin-sparing combination therapy	3	576	0.99	0.26–3.01	0.73	87	<0.001	9.38
Carbapenem-containing combination therapy vs. Carbapenem-sparing combination therapy	4	879	1.13	0.78–2.97	0.97	45	0.71	15.92

#### Subgroup Analysis on Microbiological Outcomes

Because heterogeneity existed among the analysis conducted on the overall or pooled microbiological success, we performed subgroup analysis to check the source of the heterogeneity (Table 4). There is a significant difference between the microbiological success rates among CpKP patients treated with monotherapy compared to those treated with combination therapy. Patients treated with combination therapy had more success rate (OR = 3.78, 95%*CI* = 0.95 = 6–17.9, *p*-value= <0.001, 4 studies, Number of patients = 121). No significant difference existed between the microbiological success rate among the following subgroups: 60years and above patients vs below 60years (OR = 2.89, 95%*CI* = 0.57–7.13, *p*-value = 0.45, 2 studies, Number of patients = 96), triple therapy vs. double therapy (OR = 1.29, 95%*CI* = 0.89–2.96, *p*-value = 0.24, 2 studies, Number of patients = 85), multicenter vs single center studies (OR = 2.67, 95%*CI* = 0.34–1.46, *p*-value = 0.48, 5 studies, Number of patients = 136), and prospective vs. retrospective studies (OR = 0.71, 95%*CI* = 0.55–1.08, *p*-value = 0.13, 2 studies, Number of patients = 89). Comparing studies conducted in Europe to those conducted elsewhere, it was realized that those in Europe had higher microbiological success rate as compared to those conducted elsewhere (OR = 1.99, 95%*CI* = 0.18–2.84, *p*-value = 0.03, 3 studies, Number of patients = 102). Similar to the mortality rate, the microbiological success rates were significantly different among patients treated with carbapenem vs aminoglycosides (OR = 2.07, 95%*CI* = 0.84–9.41, *p*-value = 0.06, 2 studies, Number of patients = 84) but non-significant among those treated with polymyxin vs tigecycline (OR = 3.45, 95%*CI* = 0.93–9.64, *p*-value = 0.84, 3 studies, Number of patients = 103) (Table 4). Combination therapies that contain aminoglycosides significantly increased the microbiological success rates among patients infected with CpKP as compared with CpKP patients who were treated with combination therapies which did not contain aminoglycosides (OR = 2.98, 95%*CI* = 0.98–9.13, *p*-value = 0.03, 3 studies, Number of patients = 109).

**TABLE 4:** Subgroup analysis on microbiological outcomes.

Microbiological success among	Studies	Number of patients	Test of association			Test of heterogeneity		
			*OR*	95%*CI*	*p-value*	*I* ^*2*^_*(%)*_	*p-value*	*X* ^*2*^
Combination therapy vs. Monotherapy	4	121	3.78	0.96–17.9	<0.001	89	0.89	42.17
60 years and above vs. Below 60 years	2	96	2.89	0.57–7.13	0.45	38	0.06	9.86
Triple therapy vs. Double therapy	2	85	1.29	0.89–2.96	0.24	65	0.09	3.67
Multi-center vs. Single center	5	136	2.67	0.34–1.46	0.48	<25	0.37	21.34
Data published 2017 and beyond vs. Data published below 2017	3	107	2.89	0.12–8.33	0.67	59	<0.001	35.12
Studies conducted in Europe vs. Others	3	102	1.99	0.18–2.84	0.03	68	<0.001	46.85
Prospective study vs. Retrospective study	2	89	2.17	0.79–2.15	0.38	86	0.19	6.87
Polymyxin vs. Tigecycline	3	103	3.45	0.93–9.64	0.84	71	<0.001	28.98
Carbapenem vs. Aminoglycoside	2	84	2.07	0.84–9.41	0.06	82	<0.001	46.73
Carbapenem vs. Polymyxin	1	—	—	—	—	—	—	—
Carbapenem vs. Tigecycline	0	—	—	—	—	—	—	—
Aminoglycoside-containing combination therapy vs. Aminoglycoside-sparing combination therapy vs.	3	109	2.98	0.98–9.13	0.03	47	0.58	32.09
Polymyxin-containing combination therapy vs. Polymyxin-sparing combination therapy	2	96	1.54	0.20–2.79	0.46	82	0.78	23.46
Carbapenem-containing combination therapy vs. Carbapenem-sparing combination therapy	2	84	1.16	0.92–2.99	0.91	56	0.23	36.91

### Publication Bias and Sensitivity Analysis

**FIGURE 6:**
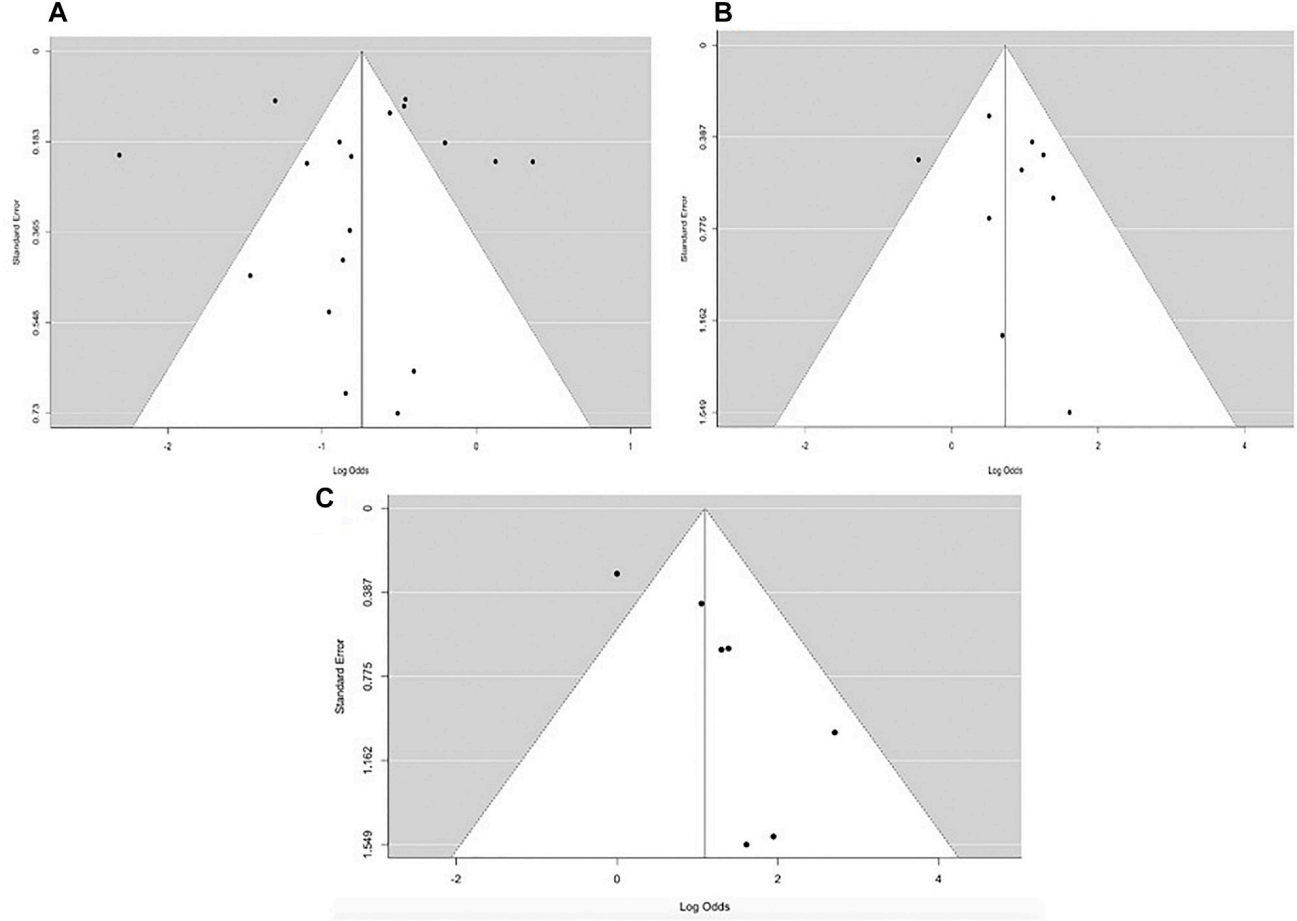
Funnel plot for overall **(A)** mortality rate **(B)** clinical success and **(C)** microbiological response.

The publication bias of the eligible papers used in our study was determined using Begg’s funnel and Egger’s test. Based on the symmetrical funnel plot for the various outcomes (Figure 6), it is evident that our results were not influenced by selection bias. Furthermore, the Egger quantitative test revealed that there was no major bias among our eligible studies, since all the pooled outcomes had *p*-values greater than 0.05 (*p* = 0.8203 for pooled mortality; *p* = 0.5942 for clinical success; and *p* = 0.189 for microbiological success). Analysis of the sensitivity or reliability of pooled estimates was then evaluated via leave-1-out sensitivity analysis. The statistical significance of the findings did not change when a specific study was omitted, demonstrating the validity and reliability of our research findings.

## Discussion

When clinicians and infection control agencies are faced with a sudden rise in infections that are caused by new antibiotic-resistant organisms such as CpKP, they turn to ask these two important questions: 1) whether there is an increase in the clinical and economic burden of the infection which is cause by this resistant organism relative to its susceptible counterpart. Knowledge on this will inform various decisions such as healthcare resource allocation, infection management and the control of these infections 2) The second question is whether treatment regimen data from clinical studies, are needed to maximize treatment-related outcomes. This particular question is very important when patients are infected with CpKP.

In this study, it was observed that tigecycline, fosfomycin and colistin recorded the highest susceptibility rate against CpKP as compared to the carbapenems which recorded almost 100% susceptibility. Bratu et al. (2005) reported that KPC-producing *K. pneumoniae* isolates are resistant to not only β-lactam antimicrobials but also aminoglycosides and fluoroquinolones. “The few remaining options are colistin, tigecycline, fosfomycin, and gentamicin, which usually harbor high MIC values, and share a suboptimal pharmacokinetic profile in terms of distribution at many infection sites (Gutiérrez-Gutiérrez et al., 2017; Poulakou et al., 2017).” Nonetheless, due to the risk of renal toxicity, the use of colistin and gentamycin has been limited. KPC-encoding bla_KPC_ gene and other drug-resistant genes carried by *K. pneumoniae* may lead to a pronounced drug resistant (Villegas et al., 2007). Drug resistance has been noted to be associated with increased mortality because patients tend to receive inappropriate empiric therapies (Tumbarello et al., 2006; Cordery et al., 2008). The High crude or pooled mortality rate realized in this study shows that CpKP has stronger invasiveness.

There was a high clinical failure as compared to higher microbiological success in this meta-analysis. The reduced clinical success realized in this study, despite a high microbiological success, reemphasizes that other clinical factors, rather than just the antibiotics (either in combination or singly), may influence treatment results. The high overall 14 days through to 90 days mortality rate (32%) observed in this meta-analysis is a cause for concern. Although the mortality rate in this study is high, it is less when compared with the results of Xu et al. (2017), who reported a mortality rate of 42.14% among infections caused by carbapenem-resistant *K. pneumoniae*. Because most of the CpKP infected patients had other comorbidities, the results obtained from the pooled mortality rate may be overestimated. Also, there may be other factors which may have contributed to this high mortality. These are: the increase risk of renal and hepatic toxicity through the use of certain medications and the increasing use of antibiotics that belong to the Polymyxin family, may have contributed to patients’ mortality (Aguayo et al., 2016); increased virulence of carbapenemase-producing strains and the fact that the use of antibiotic regimes might, in turn, increase resistance to these same medications.

The lower mortality rate associated with combination therapy as compared to monotherapy is in line with previous findings (Tzouvelekis et al., 2014; Trecarichi et al., 2016). This may be as a result of the synergistic benefit that comes with the use of combination therapies during bacterial elimination and also the ability of combination therapies to provide a broad spectra of antibacterial activity especially during polymicrobial infections. The synergistic effects of combination therapies against CpKP have been confirmed in a lot of *in vitro* studies (Poirel et al., 2016; Oliva et al., 2017). In a study by Moher et al. (2009), the synergistic activity of combination therapies on CpKP was evaluated. During Severe infections caused by CpKP, it has been recommended to use combination treatment with more than one active agent (Tumbarello et al., 2012a; Qureshi et al., 2012; Tzouvelekis et al., 2012). In a study by Tumbarello et al. (2012a), it was reported that patients who received monotherapy had a mortality rate of 54% as compared to patients who received combination therapy with the best result been obtained from those who received triple combination therapy (Tumbarello et al., 2012b). Daikos et al. (2014) reported similar findings in a study of 205 patients with Bloodstream infections caused by KPC- or VIM-producing *K. pneumoniae.*

There was no significant difference in the mortality rates when patients received double and triple antibiotic combination therapies. This result suggests that before a combination therapy would be used, there must be a broader consultation and the decision must be guided by clinical projections (e.g. comorbidity of patient, pattern of antimicrobial susceptibility, site of infection, and disease severity). It is also important to consider any possible benefits derived from increasing the number of medications, along with the likelihood of increased adverse effects associated with these antibiotics (Tamma et al., 2012). In this meta-analysis, studies conducted in Europe had lower mortality as compared with studies conducted in other continents. This may be attributable to the advance medical care and the use of different treatment strategies such as the use of double and triple antibiotic regimes, the treatment of infections with tigecycline and polymyxins, and also the use of various adjunctive procedures (e.g., catheter removal, drainage, or debridement).

Because of the high rate of resistance, poor clinical outcomes, and the fact that new drugs to effectively treat these pathogens are several years away, antimicrobial stewardship programmes must be implemented immediately at both the national (country base) and regional levels of the world to ensure the best possible patient outcomes and to preserve antimicrobials for future use. Antimicrobial stewardship programmes, among other things, can optimize antimicrobial usage, enhance patient outcomes, minimize antimicrobial resistance and health-care-associated infections, and can also reduce health-care costs. In the management of major CpKP infections, clinicians should always consult a local infectious disease expert, and the treatment should always be based on antibiotic susceptibility test and the degree of the illness. In the management, consider empiric and antibiogram-directed combination therapy for patients who are critically unwell or have deep-seated infections, but one should be mindful of the toxicities of these drugs. Also, there is a need for new, efficient and rapid detection technologies for rapid identification and detection of resistance genes such as those of the carbapenemases. This will lead to rapid identification of carbapenemase producers, allowing adequate antibiotic stewardship. This will also help prevent the development of nosocomial outbreaks caused by CpKP. The rapid detection of carbapenemase producers may also have a significant impact in preventing their spread in the community, thereby reducing the use and misuse of antimicrobials.

The strengths of this meta-analysis are that, only one strain of a particular organism was used during our search and also because we employed a high sensitive search strategy. Sensitivity analysis by repeatedly eliminating one test at a time revealed that the findings of this study were consistent, thus symbolizing the reliability of the study. The reliability and generalizability of our results was more established because of the inclusion of studies from Europe, Asia, and the Americas. Some limitations of this study are that: subgroup analysis based on clinical success and gender were not conducted due to insufficient availability of primary studies data. Also, some included studies were of only moderate quality. Majority of studies used retrospective design and therefore may be prone to selection bias. It was not possible to provide a targeted account taking into consideration all confounders, which restricts the capacity of this meta-analysis to reliably establish a causal relationship.

## Conclusion

In conclusion, there was high overall mortality and the mortality observed in CpKP-infected patients receiving combination therapy is lesser than that of those receiving monotherapy. Also, CpKP infected patients in Europe are less likely to die when compared with their counterparts in other continents. However, because most of the CpKP-infected patients had other comorbidities, the results obtained from the pooled mortality rate and the subgroup analysis may have been overestimated and one should be careful when drawing concrete conclusions from this. The choice of empirical treatment for patients with CpKP infections should be based on past treatment regimen and the antimicrobial resistant profiles of this organism in the hospital setting. Despite the advantages that combination therapies have over monotherapies, before a combination therapy would be used, there must be a broader consultation and the decision must be guided by clinical projections (e.g. comorbidity of patient, pattern of antimicrobial susceptibility, site of infection, and disease severity). New effective therapies are urgently needed to help fight infections caused by this organism. The effective use of various therapeutic options and the strict implementation of infection control measures (Poulou et al., 2012) are of utmost importance in order to prevent infections caused by CpKP. Strict national or international implementation of infection control measures and treatment guidelines will help improve healthcare, and equip governments and communities to respond to and prevent the spread of infectious diseases caused by CpKP.

## Data Availability

The raw data supporting the conclusion of this article will be made available by the authors, without undue reservation.
